# Effects of High-Velocity Spinal Manipulation on Quality of Life, Pain and Spinal Curvature in Children with Idiopathic Scoliosis: A Systematic Review

**DOI:** 10.3390/children11101167

**Published:** 2024-09-26

**Authors:** Mario Piqueras-Toharias, Alfonso Javier Ibáñez-Vera, Ana Belén Peinado-Rubia, Daniel Rodríguez-Almagro, Rafael Lomas-Vega, Ana Sedeño-Vidal

**Affiliations:** 1 Department of Health Sciences, Campus las Lagunillas, Universidad de Jaén, 23071 Jaén, Spain; mpt00016@red.ujaen.es (M.P.-T.); asedeno@ujaen.es (A.S.-V.); 2 Asociación de Fibromialgia de Jaén (AFIXA), C/Baltasar de Alcázar 5, 23008 Jaén, Spain; abpr0003@red.ujaen.es; 3 Department of Nursing, Physiotherapy and Medicine, University of Almería, La Cañada de San Urbano, 04120 Almería, Spain; dra243@ual.es

**Keywords:** scoliosis, adolescent health, spinal manipulation, chiropractic, osteopathic manipulation

## Abstract

Background/Objectives: Scoliosis is a condition that involves deformation of the spine in the coronal plane and commonly appears in childhood or adolescence, significantly limiting a person’s life. The cause is multifactorial, and treatment aims to improve the spinal curvature, prevent major pathologies, and enhance aesthetics. The objective of this review was to determine whether high-velocity low-amplitude (HVLA) spinal manipulation is more effective than other treatments for children with idiopathic scoliosis (IS). Methods: The PubMed, Web of Science, Scopus and PEDro databases were searched for both clinical trials and cohort studies. Methodological quality was assessed via the PEDro scale (for clinical trials) and the Newcastle–Ottawa scale (for observational studies). The protocol of this systematic review was registered in PROSPERO (CRD42024532442). Results: Five studies were selected for review. The results indicated moderate improvements in pain and the Cobb angle and limited improvements in quality of life. Conclusions: HVLA spinal manipulation does not seem to have significant effects on reducing spinal deformity in IS patients, nor does it significantly impact quality of life. However, this therapy may have significant effects on reducing pain in these patients.

## 1. Introduction

Idiopathic scoliosis (IS) is a progressive, three-dimensional spinal deformity that typically develops during childhood or adolescence [1]. This condition causes twisting of the spine, disrupting the normal alignment of the vertebrae, which leads to lateral displacement and rotation, ultimately resulting in an altered spinal curvature [2,3]. In accordance with the Cobb method of spine curve measurement, pathology is considered when the deviation in the coronal plane exceeds 10°. Similarly, the Scoliosis Research Society (SRS) confirmed that a diagnosis of IS is made when the Cobb angle reaches or exceeds 10° and axial rotation is evident [1,4,5]. In summary, IS is a developmental growth disorder that adversely affects the growing spine [6].

Adolescent idiopathic scoliosis (AIS) is the most prevalent type of scoliosis in children aged 10 to 18 years, accounting for 80–85% of documented cases. AIS is also characterised by an unclear aetiology that justifies the use of the term “idiopathic” [7,8]. AIS represents approximately 80% of all spinal deformities in paediatric patients, with a prevalence of 2–3% among adolescents, and it occurs 1.44 times more frequently in females than in males [9,10].

The treatment of scoliosis is influenced primarily by factors such as the patient’s development and remaining growth (which is intimately related to age and the risk of progression), the curve pattern, the severity of the deformity, and the presence or absence of other comorbidities. The amount of remaining growth is directly related to the risk of the worsening of the spinal curve, as musculoskeletal growth is greater in children. Moreover, the pubertal period is also associated with a high risk of deformity worsening, with a considerable number of cases showing significant progression [11].

There is no unified agreement on the cause of IS [1]. Many researchers believe that IS has a multifactorial origin, involving genetic, tissue-related, hormonal, biomechanical, and neurosensory factors [9]. Several studies have suggested links between IS and various conditions [12], including structural growth anomalies [6], plagiocephaly [5], melatonin receptor polymorphisms [10], alterations in calcium–phosphorus balance [13], morphological or functional abnormalities in the vestibular system [14] and orthodontic abnormalities [15] or visual impairments [5].

Spinal manipulative therapy is defined as manual therapy-based techniques in which high-velocity and low-amplitude (HVLA) forces produce a thrust that modifies the functional behaviour of a specific joint of the spine, generally increasing function and reducing pain [16]. The application of HVLA to spinal segments seems to improve the motor control of the vertebral column by stimulating the central nervous system via inputs from the paraspinal tissue mechanoreceptors that surround the joint segment [17,18]. Studies have reported that spinal manipulative therapy is commonly applied to young people for the management of musculoskeletal disorders [19]. Recent studies have evaluated the effects of spinal mobilisation on the magnitude of the curvature, trunk rotation angle and Cobb angle [3,20,21,22]. Hence, spinal manipulative therapy could be effective in reducing the sequelae of IS and improving the quality of life of patients [23]. To our knowledge, few studies have considered this technique in IS, although it appears to be free of adverse effects and effective in reducing pain. In this context, an update about its effects on IS is needed. The aim of this systematic review was to evaluate the effectiveness of manipulative spinal therapy for IS management.

## 2. Materials and Methods

### 2.1. Review Design

This systematic review was conducted following the recommendations of the Preferred Reporting Items for Systematic Reviews and Meta-Analyses (PRISMA) statement [24] and the Cochrane Handbook for Systematic Reviews of Interventions [25]. The protocol of this systematic review was registered in PROSPERO (CRD42024532442).

### 2.2. Literature Search and Bibliographical Sources

The literature search was performed using the PubMed, Web of Science (WOS), SCOPUS and PEDro databases. Two authors independently performed the literature search for relevant articles up to February 2024. In addition to the database searches, other sources, such as the reference lists of previously published studies, conference abstracts or proceedings and expert documents, were reviewed.

In terms of the search strategy, we identified two search domains, “scoliosis” and “musculoskeletal manipulations”, in accordance with the Medical Subject Headings (MeSHs) terms for MEDLINE. Synonyms and related terms were also incorporated, including “manipulation therapy”, “chiropractic manipulation”, “osteopathy”, “spinal manipulation”, “musculoskeletal manipulation”, “osteopathic medicine”, “manipulation therapy”, “osteopathic manipulative treatment”, “high-velocity manipulation” and “high-velocity low-amplitude manipulation”. The Boolean operator “AND” was used to connect the two domains, while synonyms within each domain were connected with “OR”. A combination of tags was used depending on the database (Table 1). In the search, no restrictions or filters on the publication date, language or free access to the full text were used. The titles and abstracts of all the references retrieved from the databases and additional sources were independently screened.

### 2.3. Study Selection: Inclusion and Exclusion Criteria

Two reviewers applied the criteria to reduce the risk of bias.

The studies included in our systematic review met the following inclusion criteria:

(a) Clinical trials or pilot clinical trials and cohort studies.
(b) Studies whose sample was composed of children with idiopathic scoliosis.
(c) Studies where HVLA spinal manipulation was the treatment applied in the intervention group.

The exclusion criteria were as follows:

(a) Studies analysing treatment techniques other than HVLA.
(b) Studies with mixed samples, including IS and subjects with other types of spinal curvatures.
(c) Case studies, protocols or systematic reviews.

### 2.4. Data Extraction

Two authors independently carried out the data extraction process, and any disagreements were resolved by a third author. During the data extraction process, a Microsoft Excel spreadsheet and the Mendeley platform were used to compile the data from the studies included in the systematic review. The extracted data included authorship, publication date, country, total sample size, and group size, as well as the age and sex of the participants. Data were collected on the variables of interest, the measurement tool employed and the main findings reported in each study.

### 2.5. Outcomes

The main variable assessed in this systematic review was structural deformity identified by radiographic measurement of the Cobb angle. As secondary outcome measures, we used the Scoliosis Quality of Life Index (SQLI) for quality of life, computerised noninvasive scanning by SpinalMouse for active global mobility of the spine and the visual analogue scale (VAS) for pain.

### 2.6. Methodological Quality Assessment

Two researchers were involved in measuring the risk of bias of the studies. Any discrepancies were resolved by a third researcher. The methodological quality of the studies included in this review was evaluated by the PEDro scale for clinical trials, which has been proven to be a valid and reliable tool to assess intervention studies in physiotherapy [26]. This scale comprises 11 items with two possible answers (“yes” if the criterion is met and “no” if the criterion is not met) assessing the following domains: inclusion and source criteria, random assignment, allocation concealment, baseline comparability, blinding of subjects and/or therapists and/or evaluators, follow-up greater than 85%, intention-to-treat analysis, between-group comparison and point estimates and variability [27].

The total score is calculated by adding up the responses from Items 2 to 11, obtaining a range between 0 and 10 points, which means excellent methodological quality (10–9 points), good (8–6 points), moderate (5–4 points) or poor (3 points or less) [27].

For the methodological quality assessment of the cohort studies, we used the Newcastle–Ottawa Scale (NOS). This tool considers different domains: selection of study groups, comparability of study groups and outcome assessment. The NOS has a total score between 0 and 9 and scores are classified as low quality (0–3), moderate quality (4–6) or high quality (7–9) [28].

## 3. Results

### 3.1. Study Selection

One thousand three hundred nineteen records were retrieved in the preliminary searches (*n* = 30 PubMed; *n* = 23 PEDro; *n* = 130 Scopus; *n* = 1134 Web of Science and *n* = 2 from other sources). After screening the titles/abstracts, a total of 1266 references were excluded because they were not relevant, whereas 29 articles were assessed for eligibility by applying the inclusion criteria.

**Figure 1:**
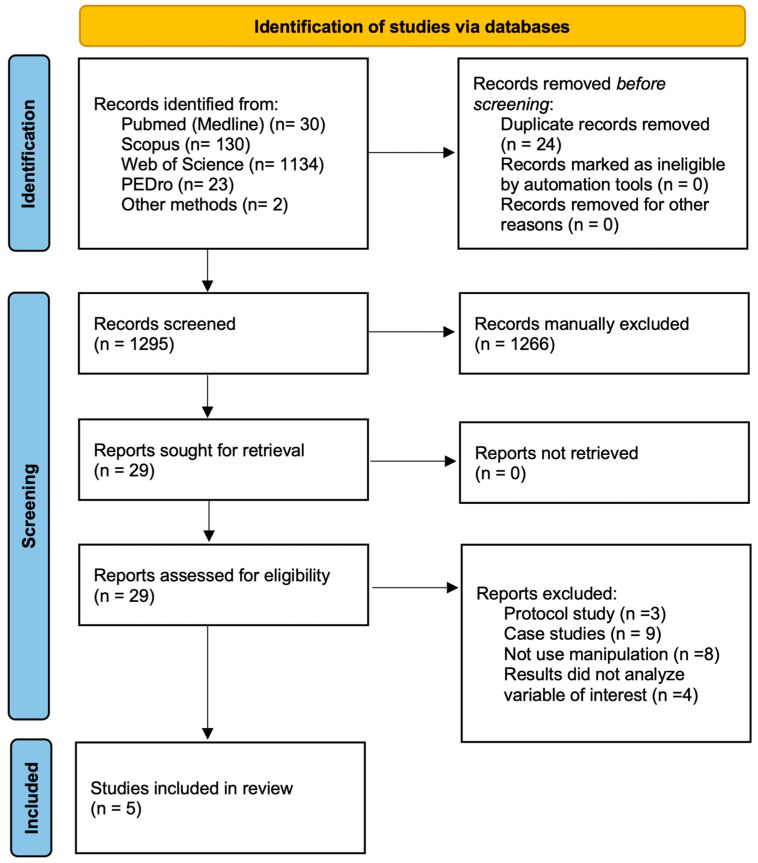
PRISMA flow diagram.

Of the twenty-nine remaining studies, twenty-four were excluded for not meeting the inclusion criteria: four of these studies were eliminated because the variables of interest were not analysed, whereas twelve studies were eliminated because of the study design and eight studies were eliminated because they did not use manipulation as the treatment. A total of five studies met the eligibility criteria and were included in the present systematic review [29,30,31,32,33]. The PRISMA flow diagram (Figure 1) shows the study selection process.

### 3.2. Methodological Quality Analysis

The methodological quality of the clinical trial studies included in this review, as evaluated with the PEDro scale, was moderate (5.5 mean score). One study was of moderate quality, and another study was of good quality (20% of the total). Table S1 shows the PEDro scale scores for the methodological quality assessment of the included studies.

The mean methodological quality of the included studies assessed with the NOS was 4 stars. Table S2 shows the NOS score for each study included.

### 3.3. Characteristics of the Studies Included in This Review

The studies included provided data from 123 participants with a mean age of 13.46 years (73.98% females; *n* = 91). The intervention group consisted of a total of 98 subjects with IS (79.59% females; *n* = 78), with a mean Cobb angle of 26.08 degrees.

The duration of the proposed interventions in each study ranged from 4 weeks to 12 months. These patients underwent spinal manipulation interventions along with orthopaedic treatment, manual soft tissue treatment and traction therapy. Among the studies included in this review, three were cohort studies, and two were randomised clinical trials that evaluated at least one of the main outcomes: structural deformity, quality of life and pain (Table 2).

### 3.4. Main Findings in the Included Studies

(a) Effects of HVLA on the structural deformity and global flexibility of the spine.

All five studies evaluated the effect of HVLA on structural deformities via the Cobb angle. Lantz et al. [29] reported that there was no discernible effect of age, initial curve severity or frequency of attention on curve severity after manipulative treatment. Rowe et al. [30] considered a posttreatment progression of <6 degrees nonsignificant and a curve improvement of between 6 and 10 degrees a significant result. They reported that the standard medical care group and the standard medical care plus sham manipulation group had no significant effects on curve improvement. The standard medical care plus chiropractic manipulation group reported clinically important improvements that were not statistically significant. Hasler et al. [31] demonstrated via repeated measurement regression analysis no therapeutic effect on the costal hump, lumbar prominence, sagittal profile or global spinal flexibility, rejecting the hypothesis that osteopathy alters trunk morphology in scoliotic girls. Post hoc analysis of the cohort by Byun et al. [32] revealed that the Cobb angle decreased markedly after 4 weeks compared with before the chiropractic technique was applied (*p* < 0.01). However, no significant difference in the Cobb angle was observed between the fourth and eighth weeks or after the eighth week. This study demonstrated that chiropractic techniques can effectively reduce the Cobb angle in the short term. Liu et al. [33] reported that after vertebral manipulation treatment, the Cobb angle was significantly lower than that before treatment (*p* < 0.01), indicating that the manipulation treatment could improve the immediate efficacy of bracing in the treatment of IS.

(b) Effects of HVLA on quality of life.

The work published by Rowe et al. [30] compared standard medical care plus chiropractic manipulation versus standard medical care plus sham manipulation and standard medical care and revealed that the improvement in the SQLI quality of life questionnaire domain scores after the intervention period was not statistically significant.

(c) Effects of HVLA on pain.

Liu et al. [33] used a similar methodology based on a brace and manipulative treatment and reported that the VAS score was significantly lower than that before treatment (*p* < 0.01).

## 4. Discussion

This review aimed to evaluate the available evidence on the effects of high-velocity, low-amplitude (HVLA) spinal manipulations on symptoms in children with idiopathic scoliosis (IS). We attempted to assess the impact of this treatment on morphological asymmetries, quality of life and pain in patients with IS treated with HVLA alone or in combination with other therapies in comparison with other treatments. The search located five studies that quantified the degrees of curvature and morphological asymmetries via radiography.

In previous studies of IS, the Cobb angle was the major prognostic and clinical indicator of curve progression. Treatment strategies that slow the progression of scoliosis, decrease the need for surgery and increase quality of life are important in the treatment of IS. In most of the studies published on HVLA spinal manipulation and IS, manipulation was not the exclusive method of treatment, which makes it difficult to determine the influence of this treatment on the results obtained in the different studies. Moreover, some of the previously published studies are case reports or case series. In relation to morphological asymmetries, in the case study carried out by Chen et al. [34], which applied a treatment based on 15 min of deep tissue massage therapy following a thoracic and bilateral lumbopelvic manipulation intervention, a decrease in the Cobb angle was observed. The study patient was a girl with a right thoracic scoliotic curve (a Cobb angle of 46 degrees). This treatment was applied twice a week for 6 weeks, followed by weekly visits for 6 weeks. After 3 months of treatment, a postintervention radiographic study revealed a decrease in the Cobb angle (to 34 degrees) of the right thoracic curvature. In addition, there was also an improvement in low back pain and the frequency of defecation. After 18 months of this treatment, the radiograph revealed an angle reduction to 30 degrees, indicating a total reduction of 16 degrees (from 46 to 30 degrees) during spinal manipulation therapy. The results of this previous study are consistent with the results shown in this review. The application of manipulative treatment together with another manual treatment makes it difficult to discern whether the change in vertebral asymmetry is due to the use of the manipulative therapy or to the set of effects caused by a global treatment. Therefore, the fact that, in almost all the selected studies [29,30,31,32,33], vertebral manipulation was not the exclusive treatment method makes it difficult to determine the influence of this therapy on the results obtained with the interventions.

On the other hand, a case report by Khauv et al. [35] reported the treatment of a 15-year-old subject diagnosed with AIS with a Cobb angle of 44 degrees, which decreased to 32 degrees after 5 months of manipulative treatment. This patient was treated with manipulative cervical treatment without any long-term effects. Similarly, Chung et al. [36] described the case of a 10-year-old girl who presented with a 35-degree thoracolumbar AIS. This patient was treated with spine manipulations for 25 weeks. The follow-up assessment via radiograph revealed a reduction of 10 degrees in the Cobb angle. In these studies, the pre- and posttreatment Cobb angle was recorded, but it was not sufficient to evaluate the percentage of cases in which improvement, stabilisation or progression of the curvature or structural deformity was observed. Nevertheless, although the studies included in this review cannot establish a causal relationship between curvature changes and manipulative treatment, they do suggest a potential association with meaningful implications for clinical practise.

Similar results regarding structural deformities were reported by Villafañe et al. [37], who described a clinical case of a 9-year-old female patient presenting a double curve pattern with a Cobb angle of 18° thoracic and 24° thoracolumbar. After 36 weeks of manipulative spinal treatment, the posttreatment Cobb angle improved the structural deformity. In any case, once again, these results must be considered with caution due to the low-quality evidence that case reports offer.

After the 2014 consensus [38,39], the SRS and the International Society on Scoliosis Orthopedic and Rehabilitation Treatment (SOSORT) indicated the need to assess results focused on patients’ quality of life impairment. From this perspective, changes in biological parameters such as the degree of curvature may not immediately lead to improvements in back pain, disability, pulmonary disorders or patients’ quality of life. For this reason, the focus on quality of life during scoliosis treatment is increasing, as is the interest in questionnaires to measure quality of life. The gold standard for clinicians regarding surgical approaches is still the Scoliosis Research Society-22 patient questionnaire (SRS-22), although its use is not as common with nonsurgical treatments.

Wang et al. [40] described a randomised controlled pilot trial with 40 subjects divided into treatment and control groups. Both groups underwent eight weeks of physiotherapy-specific scoliosis exercises. The treatment group also received spinal manipulation during the first two weeks before the exercise sessions. The spinal manipulative therapy consisted of HVLA for various segments of the spine (C7-T8 segments, lower thoracic segments, lumbar segments and the sacroiliac joint). The SRS-22 questionnaire scores were assessed at baseline and at two, four and eight weeks after the treatment. The results revealed an initial improvement in the Cobb angle and trunk symmetry, indicating that the treatment improved the quality of life of patients. The systematic review and meta-analysis conducted by Li et al. [41], which was based on whether core exercises could correct spinal deformities and improve quality of life in people with scoliosis, found significant improvements in quality of life in the core-based exercise group compared to the control group (observation, traditional treatment or bracing) as measured by the Scoliosis Research Society-22 questionnaire total score. The results showed significant between-group differences in follow-up scores and posttreatment scores. The conclusions of these previous studies are similar to those obtained in this review, showing that combined treatment that includes manipulative therapy could help reduce the Cobb angle, reduce trunk asymmetry and improve the quality of life of patients. In terms of quality of life, the clinical trial by Rowe et al. [30] included in this review concluded that spinal manipulation improved the quality of life of patients with AIS; however, the effects were only significant in terms of their mental status.

In relation to improvements in IS-related pain after manipulative treatment, in one of the studies included in our review [27], the participants showed a significant improvement in back pain, with a decrease of 2.68 points in the VAS total score (before treatment, the VAS score was 4.54 ± 1.53; after treatment, the VAS score was 1.86 ± 1.21), which was the only study that showed a favourable statistically significant result for pain. In comparison with other treatment techniques with respect to the pain in patients with IS, Atici et al. [42] demonstrated that kinesiotaping and home exercises significantly decreased the VAS scores in the treatment group of patients with Lenke type 1 AIS (before treatment, the VAS score was 6.6 ± 2; posttreatment, the VAS score was 3.5 ± 2.2). These values are similar to those of manipulative therapy, so both could be considered options for treating pain in these patients. The effectiveness of manipulative treatment in alleviating spinal pain is based on mechanisms of inhibition, which are mediated by the central nervous system [43]. Pain reduction can improve mobility and help patients improve motor performance; nonetheless, HVLA techniques can enhance the range of motion of the spinal joints and facet joints and improve muscle function. Moreover, improvements in muscle function contribute to better mobility, improved quality of life and reduced pain levels.

Finally, a comparison of our results with previously published reviews by Romano et al. [3], Théroux et al. [44], Parnell et al. [45] and Milne et al. [46] revealed inconclusive results for the use of spinal manipulation for the treatment of scoliosis and symptoms in combination with or compared to other manual treatments. The quality of evidence concerning manipulative treatments for scoliosis is low. In the previous reviews by Romano et al. [3], Parnell et al. [45] and Milne et al. [46], the number of studies included was much greater than that included in this review, possibly because these works included other manual therapies. Several previous reviews differ from ours, which does not allow us to isolate the specific effect of the manipulative treatment because they include different manual therapies. Furthermore, the work by Milne et al. [46] did not exclusively analyse the effect on IS but included other conditions, such as torticollis, asthma, otitis, infantile colic and nocturnal enuresis. In relation to the work published by Théroux et al. [38], our work includes some more updated cohort studies.

Nevertheless, this work has several limitations. First, only a few previous studies have explored the relationship between HVLA and IS. Another limitation is the low quality of the existing studies, with the primary limitation being the lack of randomised controlled trials (RCTs). Finally, another limitation was that the treatment applied to the intervention group was not homogeneous since, in addition to the manipulative therapy, they received orthopaedic treatment, manual soft tissue treatment and traction therapy. Using the best evidence available, our conclusion is based on studies with a low level of evidence and a small sample size. The methodology of the studies included in our review prevents strong conclusions from being drawn.

## 5. Conclusions

Currently, there is insufficient evidence to determine whether spinal manipulative therapy is effective at reducing pain and improving quality of life in adolescent idiopathic scoliosis patients, and there is no evidence that spinal manipulative therapy effectively reduces curve severity. The results of the included studies suggest that manipulative spinal therapy may be useful in the treatment of pain in patients with idiopathic scoliosis, but these studies presented a substantial risk of bias and low to moderate methodological quality. These results highlight the need for high-quality, randomised controlled studies of spinal manipulation in idiopathic scoliosis patients.

## Figures and Tables

**Table 1:** Search strategy used in each database.

PubMed	((spinal manipulation[Title/Abstract]) OR (musculoskeletal manipulation[Title/Abstract]) OR (manipulative therapy[Title/Abstract]) OR (manipulation therapy[Title/Abstract]) OR (osteopathic manipulative treatment[Title/Abstract]) OR (osteopathic manipulation[Title/Abstract]) OR (chiropractic manipulation[Title/Abstract]) OR (spinal adjustment[Title/Abstract]) OR (chiropractic adjustment[Title/Abstract]) OR (high-velocity manipulation[Title/Abstract]) OR (high-velocity low-amplitude manipulation[Title/Abstract])) AND ((idiopathic scoliosis[Title/Abstract]) OR (adolescent idiopathic scoliosis[Title/Abstract]))
Web of Science	((idiopathic scoliosis[Title]) AND (spinal manipulation[Title]) OR (musculoskeletal manipulation[Title]) OR (manipulative therapy[Title]) OR (osteopathic manipulative treatment[Title]) OR (osteopathic manipulation[Title]) OR (chiropractic manipulation[Title]) OR (spinal adjustment[Title]) OR (chiropractic adjustment[Title]) OR (high-velocity manipulation[Title]) OR (high-velocity low-amplitude manipulation[Title]) OR (manipulation therapy[Title]))
PEDro	Abstract and Title: adolescent idiopathic scoliosis Therapy: stretching, mobilisation, manipulation, massage Method: clinical trial When Searching: match all search terms (AND)
Scopus	((spinal manipulation[Title/Abstract]) OR (musculoskeletal manipulation[Title/Abstract]) OR (manipulative therapy[Title/Abstract]) OR (manipulation therapy[Title/Abstract]) OR (osteopathic manipulative treatment[Title/Abstract]) OR (osteopathic manipulation[Title/Abstract]) OR (chiropractic manipulation[Title/Abstract]) OR (spinal adjustment[Title/Abstract]) OR (chiropractic adjustment[Title/Abstract]) OR (high-velocity manipulation[Title/Abstract]) OR (high-velocity low-amplitude manipulation[Title/Abstract])) AND ((idiopathic scoliosis[Title/Abstract]) OR (adolescent idiopathic scoliosis[Title/Abstract]))

**Table 2:** Main characteristics of the studies included in this review.

Study	TS	Nt (F/M)	Age (Range)	Tt	W	Ses/W	Outcomes/Test	Follow-Up	MQ
Lantz et al., 2001 (United Kingdom) [29] Funding: NR	CHT	42 (26/16)	11.5 (6–17)	Manipulative treatment Postural education	1 year	2 or 3 according progression	Cobb angle (radiography)	Posttreatment	NOS score ****
Rowe et al., 2006 (EU) [30] Funding: No	RCT (pilot study)	6 (5/1)	14 (10–16)	CG: SC TG1: SC + chiropractic manipulation TG2: SC + sham manipulation	6 months	3	Cobb angle (radiography) SQLI	Posttreatment	PEDro score Moderate 5/10
Hasler et al., 2010 (Switzerland) [31] Funding: No	RCT	20 (20/0)	TG: 16.5 (15.5–18.5) CG: 14.7 (12.3–18.1)	CG: observation TG: osteopathic treatment	5	3	Cobb angle (scoliometer) Trunk morphology (video rasterstereographic surface) Spine flexibility (SpinalMouse)	Posttreatment	PEDro score Good 6/10
Byun et al., 2016 (Korea) [32] Funding: NR	CS	5 (1–4)	11.8 ± 1.3 (11–13)	Chiropractic technique Soft tissue massage	8	3	Cobb angle (radiography)	4 weeks posttreatment	NOS score ***
Liu et al., 2023 (China) [33] Funding: NR	CS	50 (39/11)	12.3 ± 2.1 (9–16)	Manipulative treatment Brace	7	1	Cobb angle (radiography) VAS	Posttreatment	NOS score ***

Abbreviations: TS, type of study; Nt, total sample size; F/M, female/male; Tt, treatment; W, weeks of treatment; Ses, sessions; VAS, visual analogue scale of pain; NR, no reference; CG, control group; TG, treatment group; SC, standard medical care (observation or brace treatment); RCT, randomised controlled trial; CT, control trial; CS, case study; CHT, cohort trial; SQLI, Scoliosis Quality of Life Index; MQ, methodological quality; NOS, Newcastle-Ottawa Scale; *, Number of stars obtained in the evaluation with NOS.

## Data Availability

Not applicable.

## Supplementary Materials

The following supporting information can be downloaded: https://www.mdpi.com/article/10.3390/children11101167/s1; Table S1: PEDro scale scores for the methodological quality assessment of the included studies; Table S2: NOS scores for the methodological quality assessment of the included studies.
