# Supplementary material for: Effects of High-Velocity Spinal Manipulation on Quality of Life, Pain and Spinal Curvature in Children with Idiopathic Scoliosis: A Systematic Review
Source: Children (Basel). 2024 Sep 26;11(10):1167. doi: 10.3390/children11101167 (PMC11506289; doi:10.3390/children11101167)
Supplement: Supplementary file 1 [file children-11-01167-s001.zip › children-3175507-supplementary.pdf]

Table S1: PEDro scale scores for the methodological quality assessment of the included studies.

Author/year	Criterion 1	Criterion 2	Criterion 3	Criterion 4	Criterion 5	Criterion 6	Criterion 7	Criterion 8	Criterion 9	Criterion 10	Criterion 11	Total	Quality
Rowe et al. 2006 [30]	Yes	Yes	Yes	No	No	No	Yes	Yes	Yes	No	No	5/10	Moderate
Hasler et al. 2010 [31]	Yes	Yes	No	Yes	No	Yes	Yes	Yes	No	Yes	No	6/10	Good

Table S2: NOS scores for the methodological quality assessment of the included studies.

Reference	Selection S1; S2; S3; S4	Comparability	Exposure E1; E2; E3	Total score
Liu et al. 2023	***	*	*	5/9
Byun and Han 2016	*	*	*	3/9
Lantz and Chen 2001	**	*	*	4/9

Each study can be awarded a maximum of one star for each numbered item within the Selection (S) and Exposure (E) categories. A maximum of two stars can be given for Comparability (C). S1, adequate case definition; S2, representativeness of the cases; S3, selection of controls; S4, definition of controls; C1, comparability of cases and controls; E1, ascertainment of exposure; E2, same method of ascertainment for cases and controls; E3, non-response rate; 1 star or 1 point.
